# Geographic Inequalities in All-Cause Mortality in Japan: Compositional or Contextual?

**DOI:** 10.1371/journal.pone.0039876

**Published:** 2012-06-27

**Authors:** Etsuji Suzuki, Saori Kashima, Ichiro Kawachi, S. V. Subramanian

**Affiliations:** 1 Department of Epidemiology, Okayama University Graduate School of Medicine, Dentistry and Pharmaceutical Sciences, Okayama, Japan; 2 Department of Public Health and Health Policy, Hiroshima University Institute of Biomedical and Health Sciences, Hiroshima, Japan; 3 Department of Society, Human Development, and Health, Harvard School of Public Health, Boston, Massachusetts, United States of America; Fundación para la Prevención y el Control de las Enfermedades Crónicas No Transmisibles en América Latina (FunPRECAL), Argentina

## Abstract

**Background:**

A recent study from Japan suggested that geographic inequalities in all-cause premature adult mortality have increased since 1995 in both sexes even after adjusting for individual age and occupation in 47 prefectures. Such variations can arise from compositional effects as well as contextual effects. In this study, we sought to further examine the emerging geographic inequalities in all-cause mortality, by exploring the relative contribution of composition and context in each prefecture.

**Methods:**

We used the 2005 vital statistics and census data among those aged 25 or older. The total number of decedents was 524,785 men and 455,863 women. We estimated gender-specific two-level logistic regression to model mortality risk as a function of age, occupation, and residence in 47 prefectures. Prefecture-level variance was used as an estimate of geographic inequalities in mortality, and prefectures were ranked by odds ratios (ORs), with the reference being the grand mean of all prefectures (value  = 1).

**Results:**

Overall, the degree of geographic inequalities was more pronounced when we did not account for the composition (i.e., age and occupation) in each prefecture. Even after adjusting for the composition, however, substantial differences remained in mortality risk across prefectures with ORs ranging from 0.870 (Okinawa) to 1.190 (Aomori) for men and from 0.864 (Shimane) to 1.132 (Aichi) for women. In some prefectures (e.g., Aomori), adjustment for composition showed little change in ORs, while we observed substantial attenuation in ORs in other prefectures (e.g., Akita). We also observed qualitative changes in some prefectures (e.g., Tokyo). No clear associations were observed between prefecture-level socioeconomic status variables and the risk of mortality in either sex.

**Conclusions:**

Geographic disparities in mortality across prefectures are quite substantial and cannot be fully explained by differences in population composition. The relative contribution of composition and context to health inequalities considerably vary across prefectures.

## Introduction

Previous studies have demonstrated the presence of geographic health inequalities between regions, between countries, and within countries [1], [2]. The bulk of studies on social and geographic inequalities in health have derived primarily from the United States and western European countries [3]–[8]. Meanwhile, although Japan has the lowest mortality in developed world, the magnitude and patterning of health inequalities within the nation remains less understood. Recently, Suzuki et al [9] examined the time-trends in social and geographic inequalities in all-cause premature adult mortality in Japan, which suggested that spatial health disparities have widened in both sexes during the decades following the collapse of the asset bubble in the early 1990s. According to this study, geographic inequalities across 47 prefectures have increased since 1995 even after adjusting for individual age and occupation in each prefecture, providing suggestive evidence of common ecologic effects of place where people live [10].

In the present study, we further examine the emerging geographic inequalities in all-cause adult mortality across prefectures in both sexes, in terms of compositional effects (i.e., effects due to the different characteristics of individuals residing in different areas) and contextual effects (i.e., effects due to features and characteristics of the area over and above the characteristics of residents) [11]. In so doing, we sought to establish whether or not the pattern of geographic inequalities in the nation is largely reflective of the variation in the composition of the areas. We hypothesized that the relative contribution of composition and context in each prefecture could substantially vary across areas, and thus the findings of the present study are expected to be very useful in providing clearer implications to mitigate the emerging geographic inequalities across prefectures. In line with most literature on area effects on health [12], we used sex, age, and occupation as a measure of composition whereas we used prefecture-level socioeconomic status as a measure of context. To provide a comprehensive perspective, the data of this study are census based and cover the whole of Japan.

## Methods

### Vital statistics and census data

Data on deaths was obtained from the *Report of Vital Statistics: Occupational and Industrial Aspects* [13], which is compiled by the Ministry of Health, Labour and Welfare every five fiscal years since 1970, coinciding with the Population Census. The latest year for which data are available is 2005. In the death notifications, respondents are asked to fill in the decedent's occupation at the time of death, and one of the following persons is obliged to submit the notification: (1) relatives who lived with the decedent, (2) other housemates, (3) landlord, estate owner, land/house agent, or (4) relatives who do not live with the decedent [14]. In 2005 fiscal year (i.e., from April 1, 2005 to March 31, 2006), occupation at the time of death was recorded for each decedent following the fourth revision of the Japan Standard Occupational Classification [15], which includes the following 11 groups: (1) specialist and technical workers, (2) administrative and managerial workers, (3) clerical workers, (4) sales workers, (5) service workers, (6) security workers, (7) agriculture, forestry and fishery workers, (8) transport and communication workers, (9) production process and related workers, (10) workers not classifiable by occupation, and (11) non-employed (a full description of each occupational group is available on-line in English [15]). Note that the group “non-employed” includes the unemployed as well as the non-labor force (e.g., home-makers, students, and the retired). Although the Census distinguishes the unemployed from home-makers, the vital records combine these categories as “non-employed.”

Denominator data for the calculation of mortality rates was obtained from the Population Census which has been conducted by the Ministry of Internal Affairs and Communications every five years since 1920 [16]. The 2005 Population Census was taken as of October 1, 2005. In the Census questionnaire, occupation was assessed by the following question [16]: “Description of work – Describe in detail the duties you are assigned to perform.” The questionnaires are delivered to every household, and one person in each household completes it on behalf of the household members. We used “production process and related workers” as the referent category because they were the largest and the second largest occupational category in men and women, respectively, excluding non-employed.

**Figure 1:**
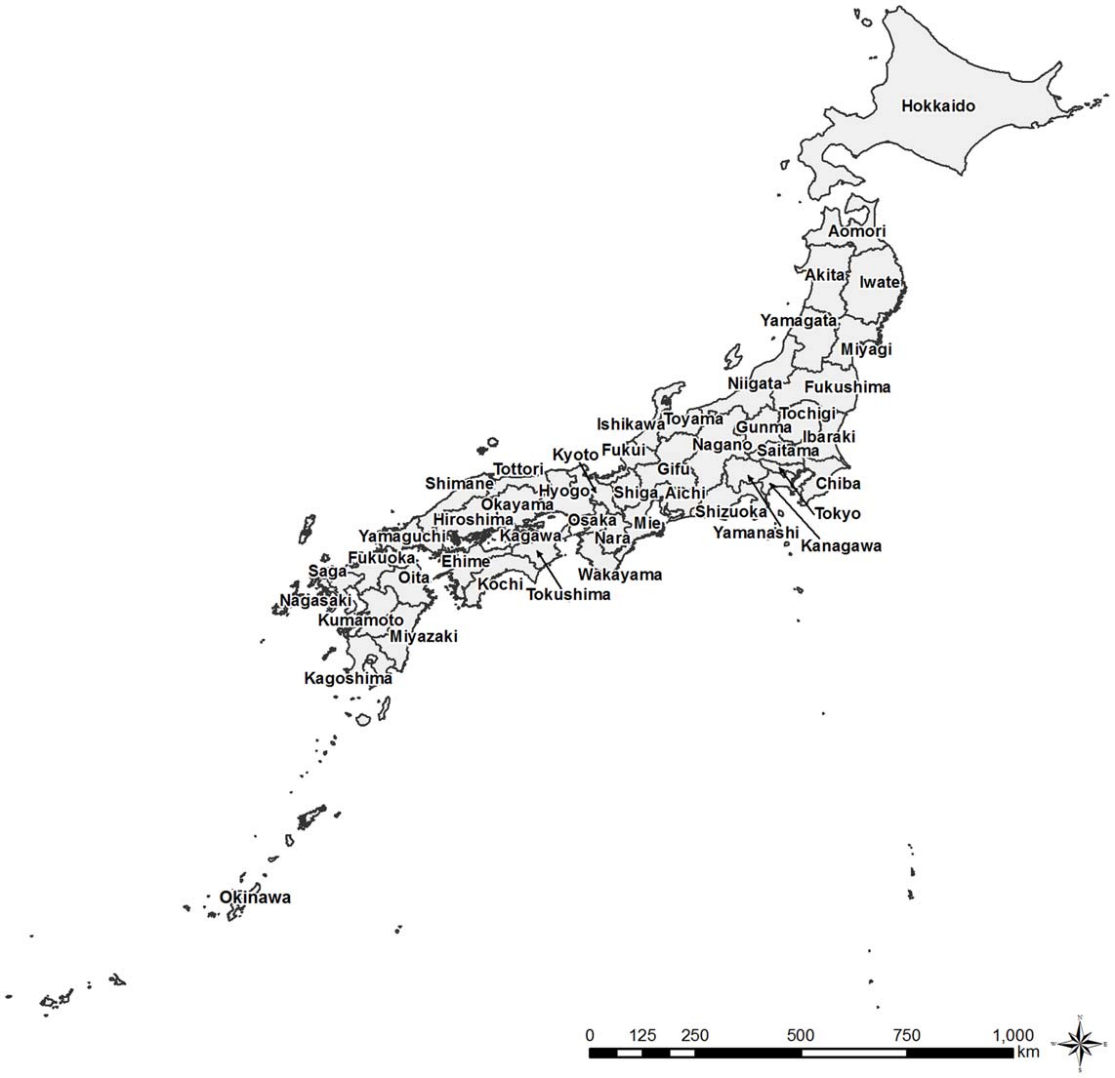
A blank map of Japan. We show the locations of 47 prefectures in Japan.

We restricted the analysis to those who are aged 25 or older to exclude students. Further, deaths records missing information on age or residence were excluded from the analysis, along with records with populations of 0 as well as cells with proportions being exceeding 1. As a result, the total number of decedents was 524,785 men and 455,863 women, in 47 prefectures (Table S1 and Figure 1).

### Measures of prefecture-level socioeconomic status

We derived prefecture-level socioeconomic status variables from the *National Survey of Family Income and Expenditure* [17], which has been conducted by the Ministry of Internal Affairs and Communications every five years since 1959. We obtained the following three variables for each prefecture from the 2004 Survey and divided them into tertiles; Gini coefficient for yearly income, average yearly income, and average savings [17]. These variables were calculated among two-or-more-person households. Although household income and savings may follow skewed distributions, median income or savings were not available.

### Statistical analysis

The data had a two-level structure of 5,687 cells for men and 5,617 cells for women at level 1, nested within 47 prefectures at level 2. Each prefecture had a maximum 121 cells (11 age groups times 11 occupational groups), and the maximum number of cells in the present data set was 5,687 (121 cells times 47) for each sex (Tables S1 and S2). We thus conducted gender-specific two-level logistic regression analysis to model mortality risk as a function of age, occupation, and residence in 47 prefectures. We used multilevel statistical procedures because of their ability to model complex variance structures at multiple levels [18]. The lowest unit of analysis was “cells,” and our models are structurally identical to models with individuals at level 1 [19].

The response variable, proportion of deaths in each cell, was modeled with allowances made for the varying denominator in each cell. We estimated a multilevel binomial logit link model, which consisted of a fixed part and a random part. Based on the results of the fixed part, we can estimate the relations between occupation and mortality, conditional on individual age variation, while the results of the random part allow estimation of prefecture-level variations in the risk of mortality. The prefecture-level variance was used as an estimate of geographic inequalities in mortality. The importance of measures of between-area variation has been emphasized for a better understanding of the socio-spatial patterning of health [12], [20]–[22].

To fit the models, we used Bayesian estimation procedures as implemented via Markov chain Monte Carlo (MCMC) methods by using MLwiN 2.25 [23], [24]. We used default diffuse priors for all the parameters, meaning that we did not favor a priori any particular values of the estimates [24]. We obtained maximum-likelihood estimates for starting values of the distribution, then 500 simulations as discarded burn-in, then 50,000 further simulations to get the distribution of interest. Based on the mean as well as the 2.5th and 97.5th percentiles of the posterior distributions, odds ratios (ORs) and 95% credible intervals (CIs) for all-cause mortality were obtained for each variable. We used the Deviance Information Criterion (DIC) to compare the goodness-of-fit of each model [24]. The DIC statistic is a combination of the fit to the data and complexity, with larger DIC values suggesting worse performance. To present the results of geographic inequalities in mortality, we created maps showing prefecture-level residuals by using ArcGIS (ESRI Japan Inc., version 10.0).

First, we examined the prefecture-level variance in mortality without including any explanatory variables as follows:
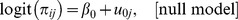
where 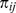 is a proportion of deaths in cell *i* in prefecture *j*. Prefecture-level random effect of the intercept (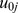) was assumed to be normally distributed with a mean of 0 and variance, 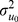. Based on the prefecture-level variance, prefectures were ranked by ORs, with the reference being the grand mean of all prefectures (value  = 1), and uncertainty was estimated by 95% CIs. Note that an estimate of the parameter 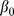 in null model represents an estimate of logarithm of the grand-mean odds for mortality among all the cell types across 47 prefectures. Then, we entered age and 11 occupations as level-1 variables as follows:



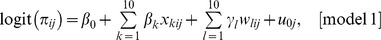
where 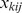 and 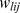 denote 10 dummy variables of age and occupation, respectively, of cell *i* in prefecture *j*. Like null model, based on the “adjusted” prefecture-level variance, prefectures were ranked by ORs, with the reference being the grand mean of all prefectures (value  = 1), and uncertainty was estimated by 95% CIs. Note that an estimate of the parameter 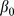 in model 1 represents an estimate of logarithm of the grand-mean odds for mortality among production process and related workers (i.e., the reference category for occupation) aged 25 to 29 years (i.e., the reference category for age) across 47 prefectures.

Subsequently, to explore the possible contextual effects by area-level deprivation, the prefecture-level socioeconomic status variable was entered into model 1 separately. Furthermore, to examine the joint effects of income inequality and average income/savings, we also entered Gini coefficient and average yearly income/savings into the model simultaneously.

We repeated these analyses by stratifying the subjects into those aged less than 65 and those aged 65 or older.

### Supplementary analyses

As a supplementary analysis, we examined occupation-specific geographic inequalities in mortality. In this analysis, following the previous report of the Population Census [25], we summarized the 11 occupations into 6 groups to increase the statistical power as follows: I. clerical, technical and managerial occupations (i.e., (1) specialist and technical workers, (2) administrative and managerial workers, and (3) clerical workers), II. sales and service occupations (i.e., (4) sales workers, (5) service workers, and (6) security workers), III. agriculture, forestry and fishery occupations (i.e., (7) agriculture, forestry and fishery workers), IV. production and transport occupations (i.e., (8) transport and communication workers and (9) production process and related workers), V. unclassifiable occupations (i.e., (10) workers not classifiable by occupation), and VI. non-employed (i.e., (11) non-employed). Then, we entered 6 prefecture-level random effect terms corresponding to the 6 aggregated occupational groups into model 1 in order to allow the fixed occupational differential on mortality to vary randomly across prefectures as follows:
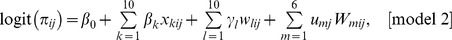
where 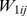, 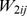, 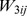, 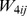, 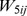, and 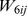 denote coding variables for clerical, technical and managerial occupations, sales and service occupations, agriculture, forestry and fishery occupations, production and transport occupations, unclassifiable occupations, and non-employed, respectively, of cell *i* in prefecture *j*. Thus, 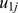, 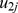, 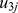, 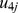, 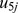, and 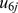 represent prefecture-level random effects among the corresponding occupations. They were assumed to be normally distributed with a mean of 0 and variances of 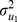, 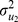, 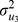, 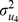, 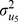, and 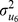, respectively. We ranked prefectures by the 6 aggregated occupational groups based on the prefecture-level occupation-specific variances.

Finally, to calculate the mean predicted probabilities of mortality for the 6 occupational groups, we removed 10 dummy variables of occupations in model 2, and entered 5 dummy variables for the 6 occupational groups as level-1 variables as follows:
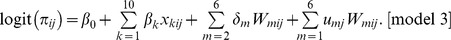



We calculated the predicted probabilities of mortality among those aged 55 to 59 because they constitute the largest population in both sexes (excluding those aged 75 or older in women). Note that, in models 2 and 3, we did not allow the intercept to vary across prefectures; rather we employed separate coding for each prefecture-level random effect term [19].

## Results

### Overall geographic inequalities in all-cause mortality

**Figure 2:**
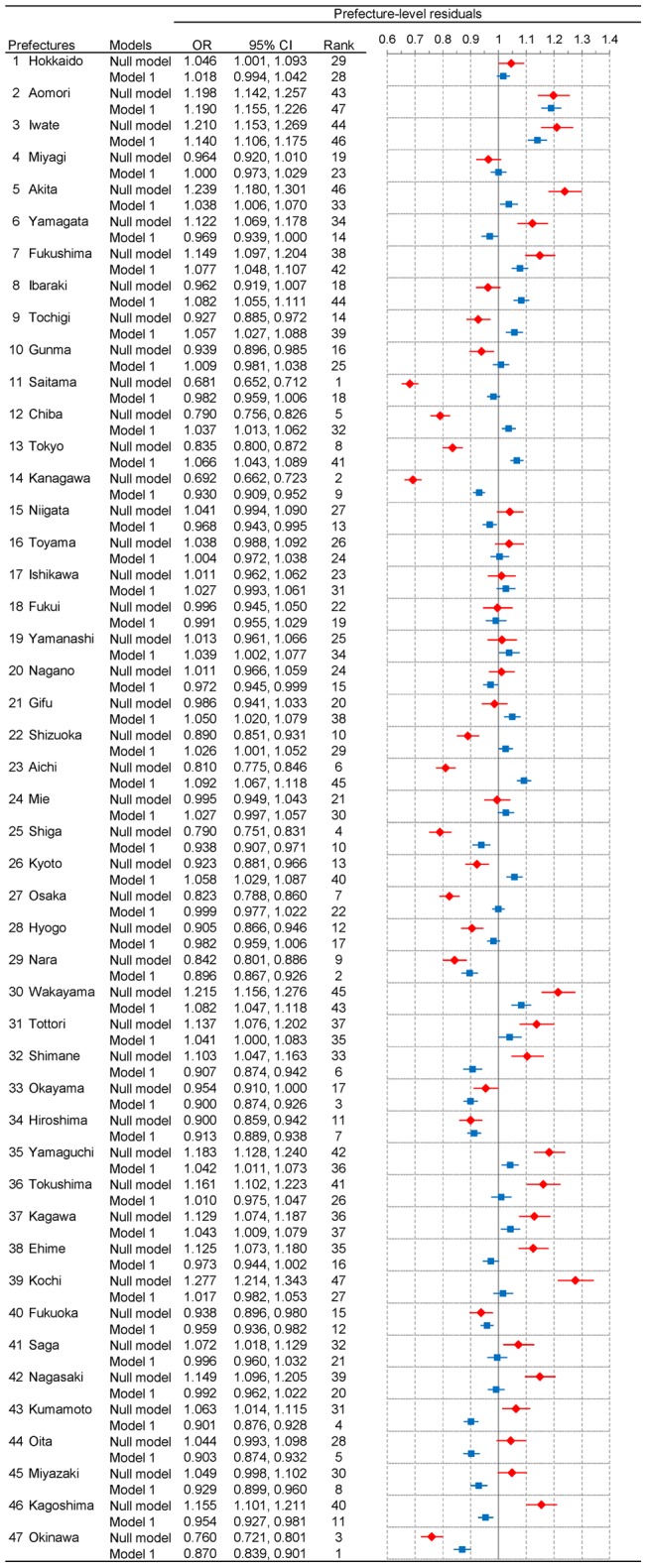
Unadjusted and adjusted prefecture-level residuals for all-cause mortality among men in 47 prefectures, Japan, 2005. Prefecture-level residuals are described in odds ratios with the reference being the grand mean of all prefectures. Red diamond and blue square represent point estimates of residuals from null model and model 1, respectively. Horizontal bars represent their 95% credible intervals. Prefectures with a lower estimate of odds for all-cause mortality are ranked higher. Note that CI and OR stand for credible interval and odds ratio, respectively.

**Figure 3:**
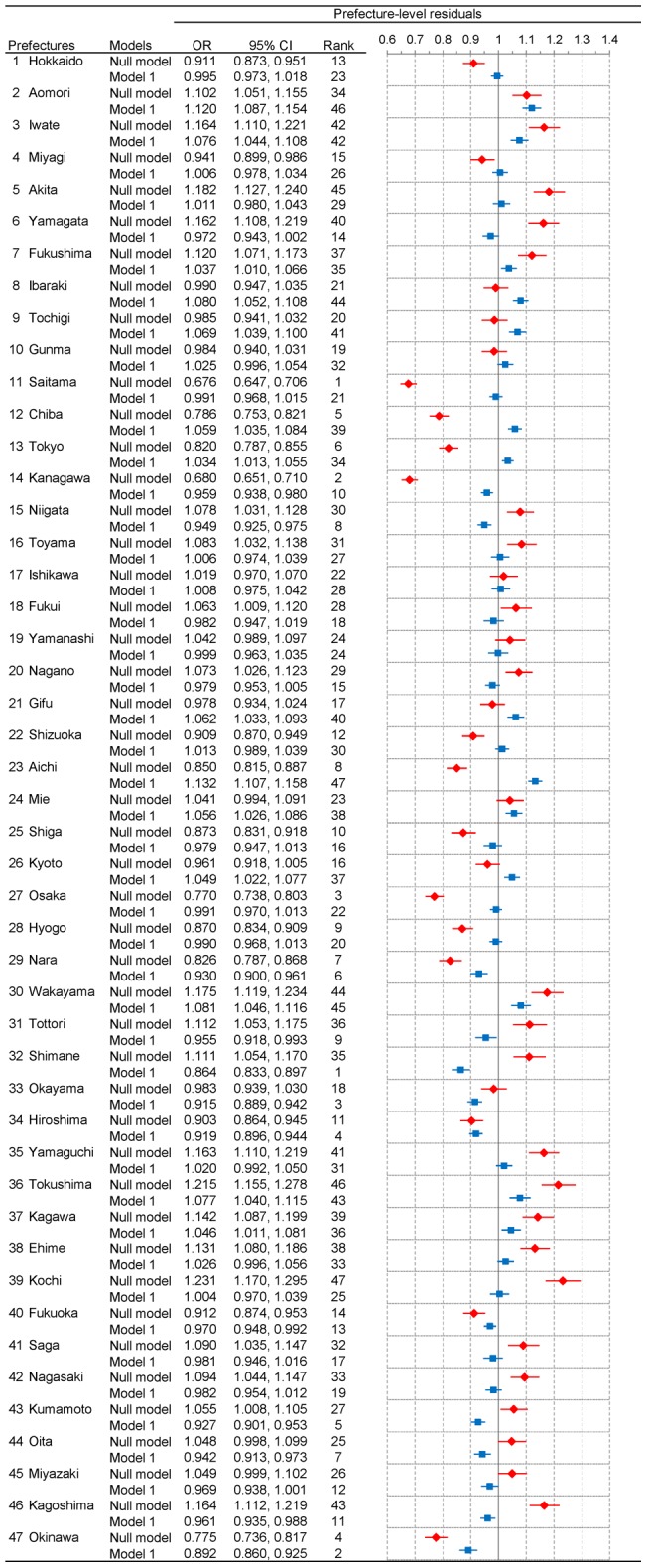
Unadjusted and adjusted prefecture-level residuals for all-cause mortality among women in 47 prefectures, Japan, 2005. Prefecture-level residuals are described in odds ratios with the reference being the grand mean of all prefectures. Red diamond and blue square represent point estimates of residuals from null model and model 1, respectively. Horizontal bars represent their 95% credible intervals. Prefectures with a lower estimate of odds for all-cause mortality are ranked higher. Note that CI and OR stand for credible interval and odds ratio, respectively.

In Figures 2 and 3, we show the results of geographic inequalities in all-cause mortality across 47 prefectures among men and women, respectively. Note that these Figures show both unadjusted and adjusted prefecture-level residuals for mortality based on the results of the random part in null model and model 1, respectively. (See Table 1 for the results of the fixed part of model 1.) Overall, the degree of geographic inequalities was more pronounced in null model (see red diamonds in Figures 2 and 3). In null model, estimates of variances of the intercepts for men and women were 0.025 (standard error (SE): 0.005) and 0.023 (SE: 0.005), respectively, and unadjusted prefecture-specific ORs for mortality ranged from 0.681 (95% CI: 0.652, 0.712) in Saitama (No. 11) to 1.277 (95% CI: 1.214, 1.343) in Kochi (No. 39) for men and from 0.676 (95% CI: 0.647, 0.706) in Saitama (No. 11) to 1.231 (95% CI: 1.170, 1.295) in Kochi (No. 39) for women. By contrast, when we adjusted for the composition (i.e., age and occupations) of each prefecture in model 1, estimates of variances of the intercepts were substantially reduced in both sexes; 0.005 (SE: 0.001) and 0.004 (SE: 0.001) among men and women, respectively. Adjusted prefecture-specific ORs ranged from 0.870 (95% CI: 0.839, 0.901) in Okinawa (No. 47) to 1.190 (95% CI: 1.155, 1.226) in Aomori (No. 2) for men and from 0.864 (95% CI: 0.833, 0.897) in Shimane (No. 32) to 1.132 (95% CI: 1.107, 1.158) in Aichi (No. 23) for women (see blue squares in Figures 2 and 3).

**Table 1:** Odds ratios for all-cause mortality associated with fixed parameters, along with the Deviance Information Criterion, Japan, 2005.

	Men					Women			
	Model 1^a^		Model 2^b^			Model 1^a^		Model 2^b^	
Characteristics	OR	95% CI	OR		95% CI	OR	95% CI	OR	95% CI
Age (y)									
25–29	1.00	Reference		1.00	Reference	1.00	Reference	1.00	Reference
30–34	1.44	1.37, 1.51		1.42	1.35, 1.50	1.45	1.35, 1.55	1.43	1.32, 1.55
35–39	2.11	2.02, 2.21		2.10	2.00, 2.20	2.01	1.88, 2.15	1.98	1.84, 2.14
40–44	3.08	2.95, 3.22		3.04	2.91, 3.18	3.28	3.08, 3.50	3.24	3.00, 3.49
45–49	4.68	4.49, 4.88		4.59	4.40, 4.79	4.78	4.50, 5.08	4.72	4.39, 5.07
50–54	7.36	7.08, 7.64		7.20	6.91, 7.50	6.99	6.60, 7.39	6.90	6.44, 7.40
55–59	11.60	11.18, 12.04		11.42	10.98, 11.88	11.24	10.64, 11.88	11.11	10.38, 11.90
60–64	10.15	9.78, 10.53		10.03	9.65, 10.43	12.73	12.06, 13.45	12.60	11.77, 13.48
65–69	11.80	11.37, 12.25		11.68	11.23, 12.14	17.16	16.26, 18.12	16.98	15.87, 18.17
70–74	18.36	17.70, 19.05		18.18	17.50, 18.90	30.24	28.67, 31.90	29.93	28.00, 32.00
≥75	57.39	55.35, 59.51		56.91	54.79, 59.11	153.32	145.45, 161.62	151.79	142.07, 162.18
Occupation									
Specialist and technical workers	3.16	3.09, 3.23	3.26		3.14, 3.40	3.28	3.13, 3.44	3.37	3.02, 3.77
Administrative and managerial workers	3.20	3.11, 3.28	3.27		3.13, 3.41	7.96	7.53, 8.43	8.21	7.30, 9.23
Clerical workers	0.96	0.93, 0.99	0.99		0.95, 1.04	0.97	0.92, 1.02	1.00	0.89, 1.12
Sales workers	1.69	1.65, 1.74	1.77		1.71, 1.84	2.05	1.96, 2.15	2.08	1.90, 2.28
Service workers	4.05	3.95, 4.16	4.24		4.09, 4.40	2.43	2.32, 2.54	2.47	2.26, 2.70
Security workers	1.78	1.70, 1.86	1.84		1.74, 1.94	16.27	14.18, 18.66	16.57	14.15, 19.40
Agriculture, forestry and fishery workers	3.33	3.26, 3.41	3.05		2.88, 3.24	2.31	2.21, 2.41	2.34	2.08, 2.63
Transport and communication workers	1.80	1.75, 1.86	1.81		1.75, 1.87	12.63	11.34, 14.08	12.57	11.26, 14.03
Production process and related workers	1.00	Reference	1.00		Reference	1.00	Reference	1.00	Reference
Workers not classifiable by occupation	7.75	7.53, 7.98	8.34		6.60, 10.53	10.49	9.96, 11.04	11.35	8.93, 14.42
Non-employed c	9.98	9.81, 10.16	9.08		8.56, 9.63	6.86	6.62, 7.12	7.03	6.24, 7.91
Deviance Information Criterion	78,803.48		74,117.36			50,873.53		49,658.28	

CI; credible interval, OR; odds ratio.

a We entered age and occupation as level-1 fixed parameters, by allowing the intercept to vary.

b We entered age and occupation as level-1 fixed parameters. Instead of allowing the intercept to vary, we entered 6 level-2 error terms corresponding to the 6 aggregated occupational groups (i.e., I. clerical, technical and managerial occupations, II. sales and service occupations, III. agriculture, forestry and fishery occupations, IV. production and transport occupations, V. unclassifiable occupations, and VI. non-employed).

c Non-employed includes the unemployed as well as the non-labor force.

When adjusting for age and occupations in model 1, almost all of the prefecture-level residuals moved toward the null (i.e., OR  = 1). We should note that the degree of change varied substantially across 47 prefectures. In some prefectures, adjustment for age and occupation yielded little change in ORs, while other prefectures exhibited striking changes. For example, as noted above, Saitama ranked at the top in null model with more than 30% lower odds for mortality in both sexes, whereas Kochi ranked at the bottom with more than 20% higher odds for mortality in both sexes. However, once we adjusted for their composition in model 1, the point estimates of ORs became close to 1, and none of them were statistically significant. In other words, Saitama and Kochi were seemingly the best and the worse prefectures, respectively, in terms of the risk for all-cause mortality, which is likely due to their composition, not context.

**Figure 4:**
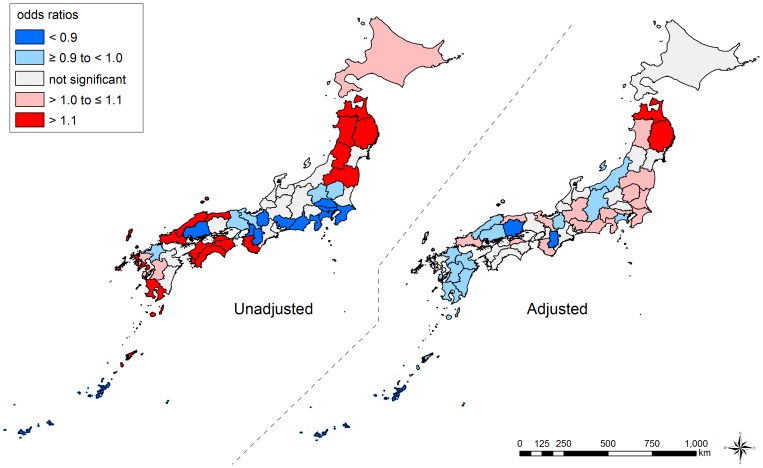
Unadjusted and adjusted geographic inequalities in all-cause mortality among men, Japan, 2005. We show the overall geographic inequalities in all-cause mortality across 47 prefectures among men. Unadjusted and adjusted inequalities were estimated from null model and model 1, respectively. Prefecture-level residuals are described by odds ratios, with the reference being the grand mean of all prefectures. Prefectures with lower odds for mortality are blue, and those with higher odds are red. The prefectures with non-significant residuals are gray.

**Figure 5:**
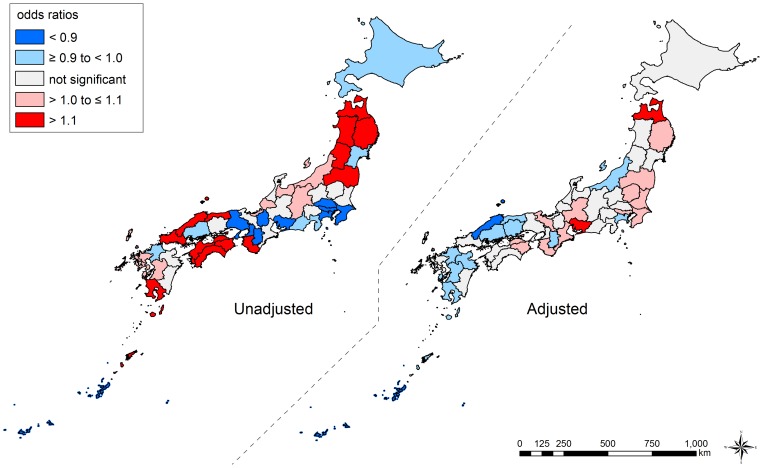
Unadjusted and adjusted geographic inequalities in all-cause mortality among women, Japan, 2005. We show the overall geographic inequalities in all-cause mortality across 47 prefectures among women. Unadjusted and adjusted inequalities were estimated from null model and model 1, respectively. Prefecture-level residuals are described by odds ratios, with the reference being the grand mean of all prefectures. Prefectures with lower odds for mortality are blue, and those with higher odds are red. The prefectures with non-significant residuals are gray.

Notably, we observed qualitative changes of ORs in some prefectures – from significantly higher ORs to significantly lower ORs, and vice versa. For example, among men, the ORs in Shimane (No. 32), Kumamoto (No. 43), and Kagoshima (No. 46) were significantly high when we did not adjust for the composition in each prefecture (null model) while they became significantly low adjusting for their composition (model 1). By contrast, in Tochigi (No. 9), Chiba (No. 12), Tokyo (No. 13), Shizuoka (No. 22), Aichi (No. 23), and Kyoto (No. 26), the pattern was reversed. The results of geographic inequalities among men and women are also shown by using maps in Figures 4 and 5, respectively.

Figures S1 and S2 show the patterns of age-stratified geographic inequalities among men and women, respectively. Overall, the patterns were relatively similar between the age groups when we adjusted for compositions in each prefecture (model 1) although we observed qualitative changes between age groups in some prefectures; for example, we observed significantly low odds for mortality among those aged less than 65 in both sexes in Chiba (No. 12) whereas we observed significantly high odds for mortality among those aged 65 or older in both sexes.

### Contextual effects by prefecture-level socioeconomic status

Overall, we found little evidence of the association between prefecture-level socioeconomic status and the risk of mortality in both sexes, conditional on individual age and occupation (Table 2). When we stratified the subjects by age, however, there was a suggestion of an inverse association between average savings and mortality among men aged less than 65 (Table 3). No clear patterns were observed for other indicators of prefecture-level socioeconomic status. When we examined the joint effects of income inequalities and average income/savings, no substantial changes was observed (data not shown).

**Table 2:** Odds ratios for all-cause mortality associated with prefecture-level socioeconomic status variables, Japan, 2005^a^.

	Men		Women	
	OR	95% CI	OR	95% CI
Gini coefficients for yearly income^b^				
Low	1.00	Reference	1.00	Reference
Middle	0.98	0.94, 1.02	0.96	0.92, 1.00
High	0.98	0.94, 1.03	0.98	0.94, 1.02
Average yearly income^b^				
High	1.00	Reference	1.00	Reference
Middle	0.97	0.92, 1.02	0.96	0.92, 1.00
Low	0.99	0.94, 1.04	0.97	0.93, 1.01
Average savings^b^				
High	1.00	Reference	1.00	Reference
Middle	1.00	0.95, 1.06	0.99	0.95, 1.03
Low	0.99	0.94, 1.04	0.98	0.94, 1.02

CI; credible interval, OR; odds ratio.

a These odds ratios were adjusted for age and occupations. Prefecture-level variables were adjusted for separately.

b These variables were calculated among two-or-more-person households.

**Table 3:** Odds ratios for all-cause mortality associated with prefecture-level socioeconomic status variables when stratified by age, Japan, 2005^a^.

	Men				Women			
	Less than 65		65 or older		Less than 65		65 or older	
	OR	95% CI	OR	95% CI	OR	95% CI	OR	95% CI
Gini coefficients for yearly income^b^								
Low	1.00	Reference	1.00	Reference	1.00	Reference	1.00	Reference
Middle	0.99	0.91, 1.07	0.97	0.92, 1.02	0.99	0.91, 1.07	0.96	0.92, 1.01
High	0.98	0.91, 1.06	0.98	0.93, 1.03	1.01	0.92, 1.10	0.98	0.94, 1.02
Average yearly income^b^								
High	1.00	Reference	1.00	Reference	1.00	Reference	1.00	Reference
Middle	0.98	0.90, 1.07	0.96	0.91, 1.01	0.96	0.88, 1.05	0.96	0.92, 1.00
Low	1.03	0.95, 1.11	0.97	0.93, 1.01	1.04	0.96, 1.13	0.97	0.93, 1.01
Average savings^b^								
High	1.00	Reference	1.00	Reference	1.00	Reference	1.00	Reference
Middle	1.03	0.95, 1.11	1.00	0.95, 1.05	1.00	0.92, 1.09	1.00	0.96, 1.05
Low	1.09	1.01, 1.17	0.98	0.93, 1.03	1.05	0.96, 1.14	0.98	0.94, 1.02

CI; credible interval, OR; odds ratio.

a These odds ratios were adjusted for age and occupations. Prefecture-level variables were adjusted for separately.

b These variables were calculated among two-or-more-person households.

### Geographic inequalities in all-cause mortality by occupational groups

Based on the results of the random part in model 2, Table 4 shows variations in all-cause mortality across 47 prefectures by the 6 aggregated occupational groups. (See Table 1 for the results of the fixed part of model 2. The DIC values of model 2 were smaller than those of model 1 in both sexes, suggesting better fit to the data.) In both sexes, unclassifiable occupations had the highest variation, and the variations among non-employed were close to 0. Among men, the variation was higher in non-manual workers (i.e., I. clerical, technical and managerial occupations and II. sales and service occupations) than manual workers (i.e., III. agriculture, forestry and fishery occupations and IV. production and transport occupations), whereas the pattern was reversed among women. Overall chi-squared values of the random parts in model 2 for men and women were 82.375 (21 degrees of freedom, *P*<0.01) and 70.504 (21 degrees of freedom, *P*<0.01), respectively.

**Table 4:** Variation in all-cause mortality between 47 prefectures by occupation groups, Japan, 2005^a^.

	Men			Women		
	Residuals on logit scale		Range of OR	Residuals on logit scale		Range of OR
Occupation	Estimate	95% CI		Estimate	95% CI	
Clerical, technical and managerial occupations	0.038	0.021, 0.055	0.666 to 1.357	0.016	0.007, 0.024	0.796 to 1.303
Sales and service occupations	0.044	0.024, 0.063	0.576 to 1.387	0.027	0.013, 0.041	0.722 to 1.326
Agriculture, forestry and fishery occupations	0.023	0.012, 0.034	0.751 to 1.408	0.055	0.028, 0.082	0.591 to 1.403
Production and transport occupations	0.031	0.017, 0.044	0.638 to 1.350	0.055	0.023, 0.086	0.681 to 1.609
Unclassifiable occupations	0.550	0.306, 0.795	0.201 to 4.454	0.515	0.279, 0.751	0.270 to 5.398
Non-employed^b^	0.005	0.003, 0.008	0.850 to 1.174	0.004	0.002, 0.006	0.862 to 1.126

CI; credible interval, OR; odds ratio.

a These variations were calculated from model 2. All the differential tests of the variations were statistically significant, except for clerical, technical and managerial occupations vs. sales and service occupations among men (*P* = 0.318), clerical, technical and managerial occupations vs. agriculture, forestry and fishery occupations among men (*P* = 0.126), clerical, technical and managerial occupations vs. production and transport occupations among men (*P* = 0.278), sales and service occupations vs. agriculture, forestry and fishery occupations among men (*P* = 0.058), agriculture, forestry and fishery occupations vs. production and transport occupations among men (*P* = 0.377), sales and service occupations vs. agriculture, forestry and fishery occupations among women (*P* = 0.067), sales and service occupations vs. production and transport occupations among women (*P* = 0.050), and agriculture, forestry and fishery occupations vs. production and transport occupations among women (*P* = 0.996).

b Non-employed includes the unemployed as well as the non-labor force.

In Tables S3 and S4, we show the rankings of 47 prefectures by the occupational groups among men and women, respectively. The corresponding patterns of geographic inequalities are also illustrated using maps in Figures S3 and S4, respectively. Table S5 shows the results of prefecture-level variance and covariance among the 6 occupational groups. Overall, men and women revealed a similar pattern (see signs of the covariances). In both sexes, the correlation coefficients between I. clerical, technical and managerial occupations and II. sales and service occupations were high (0.944 and 0.856 in men and women, respectively), and the correlation coefficients between II. sales and service occupations and IV. production and transport occupations were also high (0.911 and 0.777 in men and women, respectively). Although we observed a strong correlation between I. clerical, technical and managerial occupations and IV. production and transport occupations among men (i.e., 0.807), we did not observe this pattern among women (i.e., 0.538).

See Table S6 for the predicted number of all-cause mortality for each occupational group among those aged 55 to 59, which was calculated from the results of model 3.

## Discussion

To examine geographic inequalities in all-cause mortality in Japan, we used the 2005 vital statistics and census data. The present findings demonstrate the presence of substantial geographic variations in both sexes across 47 prefectures, even after adjusting for the composition (i.e., age and occupation) of each prefecture. Adjusting for age and occupation, ORs for all-cause mortality ranged from 0.870 in Okinawa to 1.190 in Aomori in men, while they ranged from 0.864 in Shimane to 1.132 in Aichi in women. In other words, even when taking into account the differentials of compositions in each prefecture, the risk for all-cause mortality varied by as much as 30% across prefectures. Subsequently, we used three different, but related prefecture-level socioeconomic status variables to examine their possible contextual effects – Gini coefficients for yearly income, average yearly income, and average savings. Although there was an indication of an inverse association between average savings and mortality among men aged less than 65 years, no clear patterns were observed for other prefecture-level variables. The patterns of geographic inequalities were relatively similar between non-manual occupations and production and transport occupations, primarily among men.

Previous studies from Japan have analyzed geographic inequalities in health by examining the relationship between area-level socioeconomic status and health outcomes in the corresponding areas, such as life expectancy and age-adjusted mortality rates [26]–[33]. These ecologic studies would be useful to document and monitor inequalities in health, showing the possible relationship between area-level deprivation and health. We should note, however, that the relevance of these studies is often limited since they cannot directly determine whether differences across areas are due to characteristics of the areas themselves or to differences between the characteristics of individuals residing in different areas [34]. We should also note that, due to ecologic fallacy [35], their findings cannot be necessarily extrapolated to the association between socioeconomic status and individual health.

In this study, we employed a novel multilevel approach and used the results of the random part of multilevel models to examine the geographic inequalities in all-cause mortality, by simultaneously adjusting for composition and context [18]. Indeed, rather than seeing the random part of multilevel models as a nuisance in an attempt to identify the fixed effects, estimating variance would add substantive information into the boundaries of the collectives to which individuals belong [20]–[22]. In particular, the present study would be of great use to assess the relative contribution of composition and context to the geographic inequalities across 47 prefectures. In some prefectures, adjustment for age and occupation showed little change in ORs for mortality, which implies that their composition played only a minor role. For example, adjustment for age and occupation showed little change in ORs in Aomori (No. 2) in both sexes, and they remained significantly high. This result suggests that composition matters much less than context, implying a possibility of contextual detrimental determinant(s) of health in the prefecture, e.g., economic, environmental, or social. Obviously, a possibility that this pattern emerges due to an omitted composition of the prefecture cannot be ruled out since the information about other indicators of composition (e.g., income, education, etc.) was not available. It is notable, however, that we observed substantial attenuation in ORs when adjusting for age and occupation in other prefectures. For example, in Akita (No. 5), unadjusted ORs were remarkably high by approximately 20% in both sexes, whereas they moved toward the null after adjusting for age and occupation, and OR was no longer statistically significant in women. (Note that Akita is a neighboring prefecture to Aomori as shown in Figure 1.) This finding indicates that composition in Akita played a significant role in lowering its health status in term of the risk for all-cause mortality. At the same time, the findings suggest that, once adjusting for its composition, the (unspecified) contextual effect(s) in Akita is approximately equivalent to the grand mean of all prefectures, in terms of inequalities in all-cause mortality. To summarize, based on the present findings, we can weigh the impact of composition against the impact of context on the apparent pattern of geographic inequalities, which would provide a useful clue as to direct our attention toward more effective interventions.

Notably, we observed qualitative changes before and after adjusting for age and occupation in some prefectures. In particular, in Chiba (No. 12), Tokyo (No. 13), and Aichi (No. 23), although the adjusted prefecture-level ORs for mortality were significantly high in both sexes, they were apparently “masked” by their composition in unadjusted analyses – their unadjusted ORs were remarkably low by approximately 20%. This phenomenon would be explained as a result of skewed distributions of composition(s) in these prefectures; the distribution is skewed to those who have a lower risk for mortality, which outweighs the “negatives” of the context in these prefectures (see Table 1). Notably, compared with manual workers, the risk for mortality was higher among upper non-manual workers (i.e., specialist and technical workers and administrative and managerial workers), which is different from the typical hierarchical pattern in industrialized western European and North American countries [3], [4], [6]. A recent study from Japan suggested that this remarkable pattern emerged among men following the collapse of the asset bubble in the early 1990s [9]. These discussions highlight the significance of examining the pattern of geographic inequalities in terms of composition and context, so that researchers can present its true picture.

We explored the possible contextual effects of prefecture-level socioeconomic status by using three variables. Apparently, each indicator of area socioeconomic status may be tapping into different aspects of the social environment and may be differently associated with specific health outcomes [12]. Note that we examined them after adjusting for individual age and occupation, in contrast with previous ecologic studies [26]–[33]. A previous review suggested that the studies in income inequality are more supportive in large areas, e.g., states, regions, and metropolitan areas, because in that context income inequality serves as a measure of the scale of social stratification [36]. As has been noted previously [37], a prefecture is similar to a state in the United States in terms of its population size and variations in income inequality. Although we thoroughly investigated their possible effects, no clear patterns were observed except for an inverse association between average savings and mortality among men aged less than 65 years. We should note, however, that the measures of area socioeconomic status in this study provide only truncated information about the context of areas [38]. More importantly, we lacked information at the individual level on the socioeconomic variables measured at the prefecture level, i.e., household income and household savings, which precludes a rigorous examination of true causal operation at the prefecture level. Further studies are warranted to explore contextual effects in more detail by including a sufficient number of variables measured at the individual level.

There are some limitations of this study. First, as a composition of each prefecture, only the information about sex, age, and occupation at the time of death were available. Occupations have been used as a dominant measure of socioeconomic position or occupational hazard, and researchers have been increasingly recognizing that occupation-based socioeconomic position may also reflect social networks [39]. Recent studies from Japan have indicated the significance of workplace social networks and social capital to health status among Japanese workers [40]–[42]. We should, however, note that occupations reflect only certain aspects of socioeconomic position, and in particular, the most appropriate way of defining socioeconomic position among women might not be occupation. To overcome this, we used the finest occupational classification available in the present data set, which could adjust for other omitted compositional variables (e.g., education). However, we should carefully interpret the findings among the group “non-employed” because this group included the unemployed as well as the non-labor force. Second, the smallest geographic unit available was the prefecture, and we could not explore geographic inequalities in finer detail. Although the prefecture may be a useful and valid unit of analysis since it is the unit that has direct administrative authority in the economic, education, and health sectors [43], we should note that the choice of spatial unit can lead to different conclusions regarding the pattern of geographic inequalities [12], [44], [45]. Third, a possibility of numerator/denominator bias between the two sources of information (i.e., vital statistics and census, respectively) cannot be ruled out. Although this type of measurement error may occur homogeneously across prefectures, it could exhibit varying degrees of adjustment if the person recording the notification of deaths tends to misclassify some specific occupations.

In conclusion, the results of the present study demonstrate that geographic inequalities in all-cause mortality are not simply a passive reflection of composition in each prefecture. Indeed, the present findings suggest that the relative contribution of composition and context to health inequalities substantially vary across 47 prefectures, even between neighboring prefectures. Although we should note that compositional and contextual explanations are not mutually exclusive [46]–[49], the significance of context to human health cannot be over-emphasized [34], [50]–[53], and further attention should be given to evaluating their relative contribution to the pattern of geographic inequalities in other countries. Based on the present findings, future research is needed to understand the specific determinants of emerging geographic inequalities in Japan – either compositional or contextual.

## Supporting Information

Figure S1 **Unadjusted and adjusted geographic inequalities in all-cause mortality among men, stratified by age groups, Japan, 2005.** We show the overall geographic inequalities in all-cause mortality across 47 prefectures among men. Unadjusted and adjusted inequalities were estimated from null model and model 1, respectively. Prefecture-level residuals are described by odds ratios, with the reference being the grand mean of all prefectures. Prefectures with lower odds for mortality are blue, and those with higher odds are red. The prefectures with non-significant residuals are gray.
(PDF)

Figure S2 **Unadjusted and adjusted geographic inequalities in all-cause mortality among women, stratified by age groups, Japan, 2005.** We show the overall geographic inequalities in all-cause mortality across 47 prefectures among women. Unadjusted and adjusted inequalities were estimated from null model and model 1, respectively. Prefecture-level residuals are described by odds ratios, with the reference being the grand mean of all prefectures. Prefectures with lower odds for mortality are blue, and those with higher odds are red. The prefectures with non-significant residuals are gray.
(PDF)

Figure S3 **Geographic inequalities in all-cause mortality by occupational groups among men, Japan, 2005.** We show the geographic inequalities in all-cause mortality across 47 prefectures for the six aggregated occupational groups, conditional on individual age and occupation. Prefecture-level residuals from model 2 are described by odds ratios, with the reference being the grand mean of all prefectures. Prefectures with lower odds for mortality are blue, and those with higher odds are red. The prefectures with non-significant residuals are gray.
(PDF)

Figure S4 **Geographic inequalities in all-cause mortality by occupational groups among women, Japan, 2005.** We show the geographic inequalities in all-cause mortality across 47 prefectures for the six aggregated occupational groups, conditional on individual age and occupation. Prefecture-level residuals from model 2 are described by odds ratios, with the reference being the grand mean of all prefectures. Prefectures with lower odds for mortality are blue, and those with higher odds are red. The prefectures with non-significant residuals are gray.
(PDF)

Table S1 **Description of data used for multilevel models analyzing all-cause mortality in 47 prefectures, Japan, 2005.**
(PDF)

Table S2 **Detailed description of data used for multilevel models analyzing all-cause mortality in 47 prefectures, Japan, 2005.**
(PDF)

Table S3 **Prefecture-level residuals for all-cause mortality by occupations among men, Japan, 2005.**
(PDF)

Table S4 **Prefecture-level residuals for all-cause mortality by occupations among women, Japan, 2005.**
(PDF)

Table S5 **Variance and covariance matrices of prefecture-level variances of each occupation group, Japan, 2005.**
(PDF)

Table S6 **Predicted number of all-cause mortality (per 100,000) by each occupation group, Japan, 2005.**
(PDF)
