# Supplementary material for: Geographic Inequalities in All-Cause Mortality in Japan: Compositional or Contextual?
Source: PLoS One. 2012 Jun 27;7(6):e39876. doi: 10.1371/journal.pone.0039876 (PMC3384616; doi:10.1371/journal.pone.0039876)
Supplement: Figure S1 — Unadjusted and adjusted geographic inequalities in all-cause mortality among men, stratified by age groups, Japan, 2005. We show the overall geographic inequalities in all-cause mortality across 47 prefectures among men. Unadjusted and adjusted inequalities were estimated from null model and model 1, respectively. Prefecture-level residuals are described by odds ratios, with the reference being the grand mean of all prefectures. Prefectures with lower odds for mortality are blue, and those with higher odds are red. The prefectures with non-significant residuals are gray. (PDF) [file pone.0039876.s001.pdf]

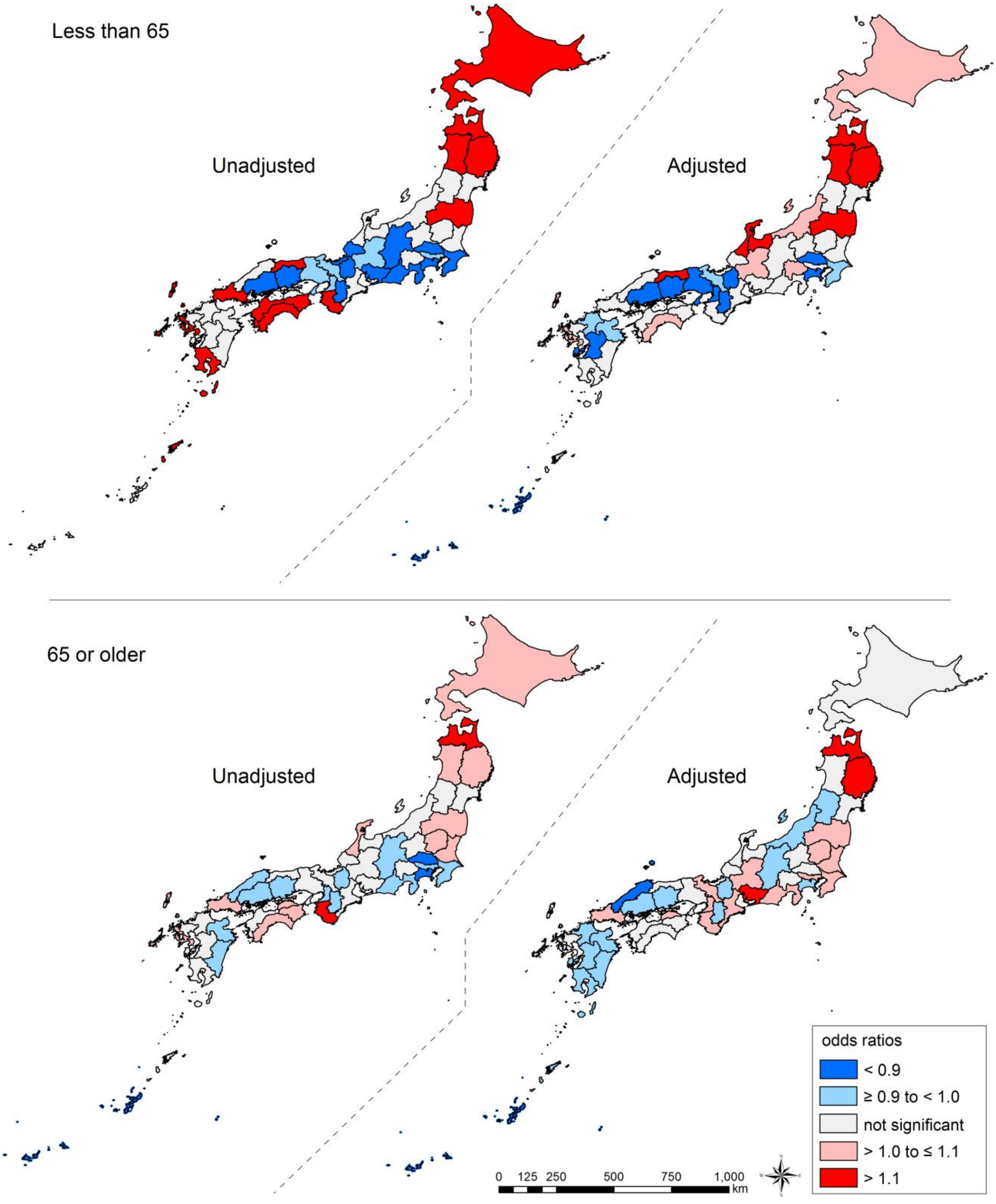

Figure S1. Unadjusted and adjusted geographic inequalities in all-cause mortality among men, stratified by age groups, Japan, 2005.
