# Supplementary material for: Geographic Inequalities in All-Cause Mortality in Japan: Compositional or Contextual?
Source: PLoS One. 2012 Jun 27;7(6):e39876. doi: 10.1371/journal.pone.0039876 (PMC3384616; doi:10.1371/journal.pone.0039876)
Supplement: Figure S3 — Geographic inequalities in all-cause mortality by occupational groups among men, Japan, 2005. We show the geographic inequalities in all-cause mortality across 47 prefectures for the six aggregated occupational groups, conditional on individual age and occupation. Prefecture-level residuals from model 2 are described by odds ratios, with the reference being the grand mean of all prefectures. Prefectures with lower odds for mortality are blue, and those with higher odds are red. The prefectures with non-significant residuals are gray. (PDF) [file pone.0039876.s003.pdf]

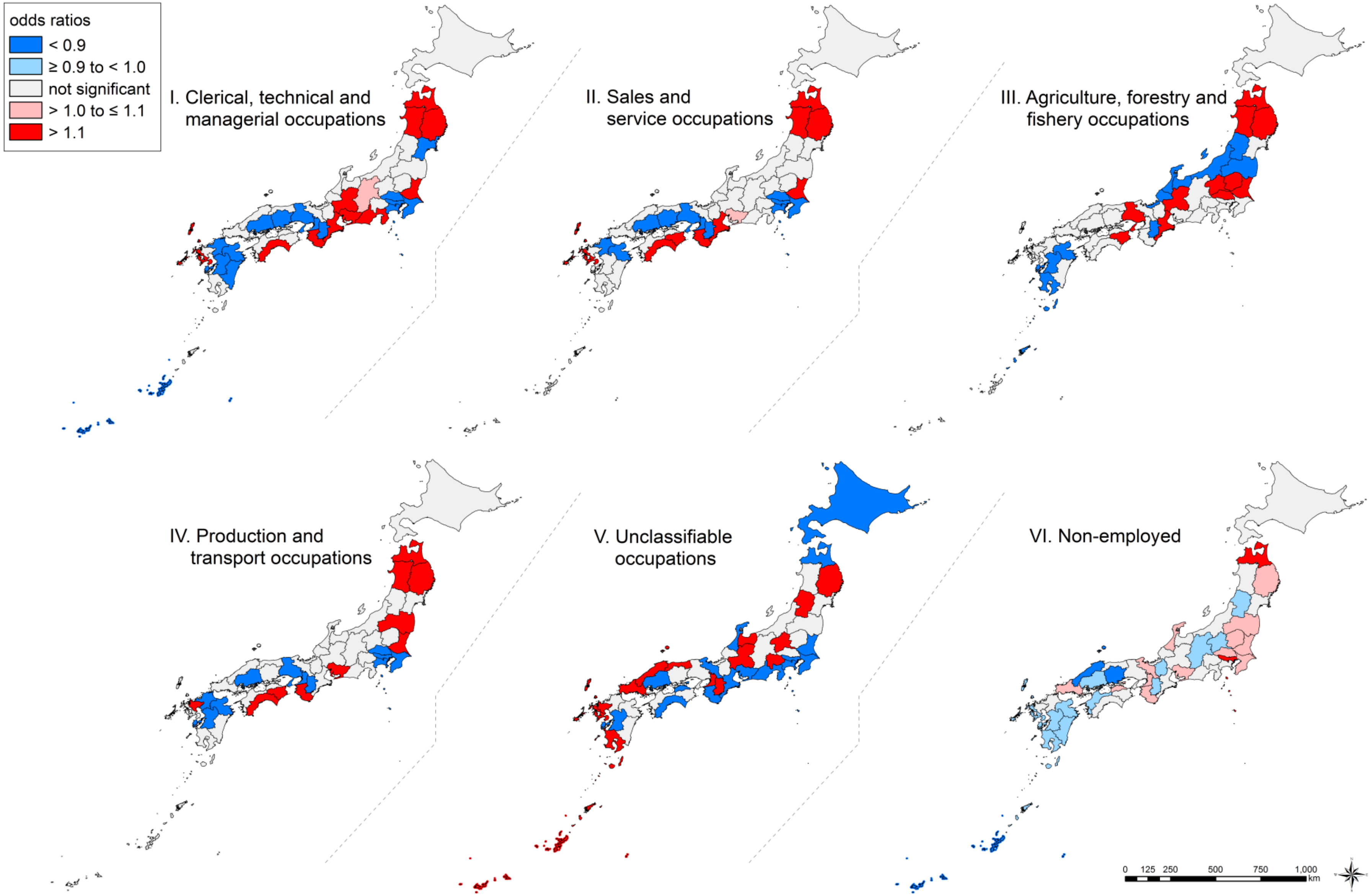

Figure S3. Geographic inequalities in all-cause mortality by occupational groups among men, Japan, 2005.
