# Supplementary material for: Geographic Inequalities in All-Cause Mortality in Japan: Compositional or Contextual?
Source: PLoS One. 2012 Jun 27;7(6):e39876. doi: 10.1371/journal.pone.0039876 (PMC3384616; doi:10.1371/journal.pone.0039876)
Supplement: Table S1 — Description of data used for multilevel models analyzing all-cause mortality in 47 prefectures, Japan, 2005. (PDF) [file pone.0039876.s005.pdf]

Table S1. Description of data used for multilevel models analyzing all-cause mortality in 47 prefectures, Japan, 2005

	Men					Women				
	No. of cells [a]	No. of deaths	Total population	Mortality rate per 100,000 [b]	(SD)	No. of cells [a]	No. of deaths	Total population	Mortality rate per 100,000 [b]	(SD)
Overall	5,687	524,785	42,461,355	1,121	(2,387)	5,617	455,863	49,002,298	1,212	(6,855)
Prefectures										
1 Hokkaido	121	25,888	1,839,355	964	(1,643)	119	20,883	2,209,893	894	(2,451)
2 Aomori	121	7,595	471,698	1,144	(1,894)	119	6,506	569,982	593	(1,746)
3 Iwate	121	7,609	467,925	1,505	(3,111)	120	6,539	542,112	1,010	(3,762)
4 Miyagi	121	9,943	765,936	989	(1,832)	121	8,487	869,501	2,165	(11,670)
5 Akita	121	6,612	397,154	1,324	(2,689)	120	5,837	476,690	1,331	(9,167)
6 Yamagata	121	6,328	419,214	1,471	(3,571)	119	5,780	480,349	2,210	(12,943)
7 Fukushima	121	10,872	703,829	1,237	(2,593)	120	9,168	789,901	2,053	(10,414)
8 Ibaraki	121	13,243	1,022,039	1,365	(2,956)	119	11,207	1,091,804	768	(2,252)
9 Tochigi	121	8,700	696,471	1,074	(1,830)	119	7,578	741,866	550	(1,166)
10 Gunma	121	8,863	700,703	1,588	(3,359)	119	7,802	764,438	852	(2,767)
11 Saitama	121	22,119	2,403,612	960	(1,795)	119	17,794	2,531,752	933	(2,342)
12 Chiba	121	21,778	2,042,374	958	(1,808)	119	17,871	2,189,457	646	(1,435)
13 Tokyo	121	45,019	3,997,259	904	(1,606)	121	37,914	4,451,317	1,625	(7,184)
14 Kanagawa	121	28,294	3,026,092	835	(1,488)	119	22,296	3,155,636	788	(2,014)
15 Niigata	121	11,890	848,598	1,002	(1,649)	120	10,659	954,218	1,324	(9,170)
16 Toyama	121	5,418	387,652	1,188	(2,321)	120	4,979	443,433	2,305	(12,905)
17 Ishikawa	121	5,334	391,839	880	(1,585)	120	4,777	452,311	1,757	(10,187)
18 Fukui	121	3,725	277,679	895	(1,560)	120	3,488	316,444	432	(1,221)
19 Yamanashi	121	4,105	301,107	1,283	(2,429)	120	3,622	335,339	1,449	(9,459)
20 Nagano	121	10,400	764,052	1,039	(1,847)	119	9,466	851,462	548	(1,398)
21 Gifu	121	9,505	716,075	1,594	(4,004)	119	8,174	806,126	955	(3,711)
22 Shizuoka	121	15,940	1,328,578	1,041	(1,805)	119	13,491	1,431,551	1,559	(9,266)
23 Aichi	121	26,966	2,467,516	1,150	(2,075)	120	22,890	2,593,716	1,995	(9,926)
24 Mie	121	8,541	637,731	1,073	(1,980)	120	7,771	720,120	1,210	(4,978)
25 Shiga	121	4,839	454,867	1,123	(2,255)	119	4,512	498,379	541	(1,430)
26 Kyoto	121	10,386	835,641	986	(1,780)	120	9,886	992,619	905	(4,671)
27 Osaka	121	31,452	2,831,377	975	(2,114)	121	26,466	3,311,228	1,880	(8,499)
28 Hyogo	121	22,132	1,814,432	901	(1,587)	120	19,330	2,140,413	1,432	(6,245)
29 Nara	121	5,205	458,776	1,129	(2,423)	119	4,762	555,966	2,279	(13,032)
30 Wakayama	121	5,679	347,700	1,029	(1,881)	119	5,201	427,111	721	(3,235)
31 Tottori	121	3,070	200,587	1,112	(1,836)	119	2,725	236,211	474	(1,203)
32 Shimane	121	3,770	253,823	1,300	(2,596)	119	3,441	298,721	963	(3,518)
33 Okayama	121	8,327	648,024	983	(2,081)	121	7,661	751,866	2,103	(11,683)
34 Hiroshima	121	11,664	961,988	867	(1,588)	120	10,358	1,105,481	1,502	(8,452)
35 Yamaguchi	121	8,033	505,320	1,181	(2,173)	121	7,369	611,531	2,299	(12,832)
36 Tokushima	121	4,211	269,598	1,282	(2,833)	119	4,045	321,197	1,466	(5,547)
37 Kagawa	121	5,251	345,630	1,034	(1,992)	119	4,728	399,618	561	(1,481)
38 Ehime	121	7,430	491,118	1,100	(1,995)	120	6,978	595,242	1,339	(5,398)
39 Kochi	121	4,527	263,744	1,020	(1,837)	120	4,139	324,308	1,056	(4,803)
40 Fukuoka	121	19,947	1,579,288	909	(1,616)	119	18,207	1,923,603	646	(1,531)
41 Saga	121	4,058	281,316	1,808	(5,754)	118	3,837	339,660	1,053	(4,553)
42 Nagasaki	121	7,497	485,149	1,351	(3,482)	119	6,788	598,707	1,044	(3,651)
43 Kumamoto	121	8,550	597,846	823	(1,671)	118	7,982	729,827	813	(2,738)
44 Oita	121	5,641	401,297	972	(1,749)	119	5,312	489,183	811	(2,425)
45 Miyazaki	121	5,353	379,148	898	(1,708)	119	4,980	457,951	604	(2,252)
46 Kagoshima	121	8,935	575,642	1,384	(3,214)	119	8,523	706,657	854	(2,063)
47 Okinawa	121	4,141	404,556	1,038	(2,101)	120	3,654	454,852	1,576	(9,480)

SD; standard deviation

[a] These cells are cross-classified by sex, age (5 year categories), and 11 occupations.

[b] Mortality rate was calculated on the basis of the means of the proportion of deaths for each prefecture across all cell types.
