# Supplementary material for: Geographic Inequalities in All-Cause Mortality in Japan: Compositional or Contextual?
Source: PLoS One. 2012 Jun 27;7(6):e39876. doi: 10.1371/journal.pone.0039876 (PMC3384616; doi:10.1371/journal.pone.0039876)
Supplement: Table S2 — Detailed description of data used for multilevel models analyzing all-cause mortality in 47 prefectures, Japan, 2005. (PDF) [file pone.0039876.s006.pdf]

Table S2. Detailed description of data used for multilevel models analyzing all-cause mortality in 47 prefectures, Japan, 2005

	Men					Women				
Characteristics	No. of cells [a]	No. of deaths	Total population	Mortality rate per 100,000 [b]	(SD)	No. of cells [a]	No. of deaths	Total population	Mortality rate per 100,000 [b]	(SD)
Overall	5,687	524,785	42,461,355	1,121	(2,387)	5,617	455,863	49,002,298	1,212	(6,855)
Level 1: cell										
Specialist and technical workers										
25-29 y	47	265	502,913	69	(40)	47	81	614,310	15	(15)
30-34 y	47	351	613,274	70	(34)	47	113	543,430	22	(18)
35-39 y	47	455	609,894	91	(43)	47	137	484,864	26	(18)
40-44 y	47	613	605,820	121	(48)	47	155	508,252	36	(22)
45-49 y	47	963	526,711	203	(70)	47	267	499,786	53	(24)
50-54 y	47	1,480	462,924	371	(117)	47	360	407,388	86	(45)
55-59 y	47	2,429	419,165	664	(188)	47	440	293,532	137	(58)
60-64 y	47	1,732	210,114	946	(317)	47	262	108,332	263	(157)
65-69 y	47	1,454	116,426	1,416	(461)	47	278	48,880	640	(361)
70-74 y	47	1,669	74,272	2,669	(813)	47	293	27,196	1,287	(622)
≥75 y	47	4,777	77,568	7,251	(1,742)	47	1,641	25,061	7,375	(2,578)
Administrative and managerial workers										
25-29 y	47	16	9,496	162	(410)	47	2	1,898	168	(1,045)
30-34 y	47	36	32,036	136	(202)	47	7	4,913	149	(482)
35-39 y	47	75	57,903	132	(134)	47	9	7,831	63	(174)
40-44 y	47	134	95,378	135	(103)	47	32	12,407	249	(403)
45-49 y	47	285	134,099	217	(120)	47	58	16,612	317	(355)
50-54 y	47	657	196,112	343	(139)	47	95	22,625	317	(311)
55-59 y	47	1,429	297,642	496	(163)	47	210	32,593	599	(396)
60-64 y	47	1,154	208,650	566	(196)	47	164	24,404	576	(517)
65-69 y	47	1,104	117,950	943	(404)	47	180	17,955	999	(895)
70-74 y	47	1,333	76,366	1,719	(597)	47	248	14,877	1,583	(948)
≥75 y	47	3,663	69,210	5,460	(1,459)	47	1,219	18,704	6,035	(2,104)
Clerical workers										
25-29 y	47	155	378,804	43	(26)	47	88	983,008	10	(11)
30-34 y	47	224	551,747	46	(27)	47	123	1,089,085	11	(10)
35-39 y	47	264	548,140	55	(25)	47	165	918,008	17	(13)
40-44 y	47	365	568,321	74	(37)	47	180	889,675	22	(15)
45-49 y	47	557	561,187	101	(48)	47	251	796,552	33	(23)
50-54 y	47	917	588,590	174	(48)	47	319	749,224	47	(20)
55-59 y	47	1,247	607,194	225	(71)	47	415	688,828	64	(26)
60-64 y	47	530	289,141	200	(112)	47	185	308,581	61	(40)
65-69 y	47	286	119,739	259	(168)	47	152	139,969	121	(98)
70-74 y	47	203	56,481	374	(276)	47	122	69,850	168	(125)
≥75 y	47	394	35,357	1,215	(589)	47	446	46,083	986	(756)
Sales workers										
25-29 y	47	153	555,127	30	(21)	47	35	351,855	11	(20)
30-34 y	47	194	740,753	29	(19)	47	65	314,121	22	(23)
35-39 y	47	263	689,206	51	(36)	47	56	282,329	23	(29)
40-44 y	47	346	630,060	66	(46)	47	99	296,912	37	(32)
45-49 y	47	595	567,370	121	(52)	47	172	315,224	55	(32)
50-54 y	47	930	565,315	195	(72)	47	280	367,940	83	(55)
55-59 y	47	1,604	620,504	297	(83)	47	481	403,573	123	(50)
60-64 y	47	1,355	347,729	460	(178)	47	390	229,178	187	(88)
65-69 y	47	1,260	199,552	732	(216)	47	349	132,749	281	(109)
70-74 y	47	1,419	119,074	1,349	(390)	47	408	84,631	483	(216)
≥75 y	47	3,636	103,961	3,792	(1,021)	47	1,919	92,571	1,980	(575)
Service workers										
25-29 y	47	208	261,378	90	(59)	47	76	347,744	23	(24)
30-34 y	47	234	228,037	118	(72)	47	95	326,844	27	(23)
35-39 y	47	258	169,690	169	(102)	47	87	330,242	31	(28)
40-44 y	47	329	138,223	275	(141)	47	148	375,230	46	(34)
45-49 y	47	567	130,897	468	(220)	47	273	409,948	70	(34)
50-54 y	47	873	155,813	629	(228)	47	440	496,370	91	(39)
55-59 y	47	1,498	192,817	837	(216)	47	701	573,042	133	(38)
60-64 y	47	1,268	164,667	894	(345)	47	507	347,727	147	(58)
65-69 y	47	1,113	111,802	1,227	(371)	47	468	166,467	274	(131)
70-74 y	47	943	52,766	1,948	(617)	47	401	68,627	596	(267)
≥75 y	47	1,947	27,980	7,462	(2,395)	47	1,447	40,862	3,320	(931)
Security workers										
25-29 y	47	48	107,718	49	(71)	47	4	9,620	22	(85)
30-34 y	47	62	107,016	58	(77)	47	4	8,635	50	(226)
35-39 y	47	84	84,845	92	(108)	47	11	4,942	163	(431)
40-44 y	47	86	93,815	99	(82)	47	6	4,269	328	(1,021)
45-49 y	47	201	104,924	197	(125)	47	16	4,244	291	(618)
50-54 y	47	272	126,450	213	(95)	47	32	4,765	736	(1,275)
55-59 y	47	448	128,129	344	(183)	47	38	4,332	1,012	(1,522)
60-64 y	47	295	79,251	380	(217)	47	30	2,351	1,138	(2,049)
65-69 y	47	217	45,496	493	(284)	46	19	964	1,372	(4,064)
70-74 y	47	133	16,122	808	(661)	42	19	352	10,409	(25,490)
≥75 y	47	210	3,995	5,757	(4,837)	13	43	75	70,310	(35,948)
Agriculture, forestry and fishery workers										
25-29 y	47	28	49,558	53	(94)	47	2	14,118	25	(123)
30-34 y	47	66	55,049	125	(142)	47	5	22,270	12	(41)
35-39 y	47	88	55,198	141	(129)	47	9	31,863	56	(194)
40-44 y	47	158	69,055	216	(148)	47	33	48,506	59	(88)
45-49 y	47	252	92,186	283	(147)	47	69	71,976	116	(157)
50-54 y	47	624	134,697	490	(214)	47	154	108,322	133	(109)
55-59 y	47	1,010	171,401	588	(158)	47	251	137,973	195	(91)
60-64 y	47	1,311	195,922	666	(198)	47	356	165,391	227	(93)
65-69 y	47	2,311	265,709	857	(212)	47	627	208,841	309	(100)
70-74 y	47	4,179	288,807	1,429	(268)	47	942	199,350	462	(113)
≥75 y	47	12,133	315,578	3,796	(697)	47	3,725	183,663	2,061	(563)
Transport and communication workers										
25-29 y	47	93	134,560	91	(87)	47	5	8,708	30	(116)
30-34 y	47	155	213,836	71	(57)	47	11	12,758	66	(182)
35-39 y	47	212	223,214	115	(78)	47	11	12,166	66	(233)
40-44 y	47	306	208,272	163	(78)	47	21	11,546	86	(195)
45-49 y	47	439	200,313	230	(88)	47	33	11,020	242	(363)
50-54 y	47	718	253,018	292	(99)	47	47	12,233	387	(543)
55-59 y	47	1,215	343,583	346	(89)	47	70	12,143	431	(546)
60-64 y	47	715	217,755	350	(152)	47	43	4,820	839	(1,110)
65-69 y	47	395	91,342	439	(260)	47	37	1,467	2,079	(3,716)
70-74 y	47	202	24,804	789	(626)	46	37	318	14,183	(25,589)
≥75 y	47	228	4,341	7,022	(5,669)	18	64	97	66,272	(33,389)
Production process and related workers										
25-29 y	47	350	1,318,325	29	(14)	47	27	312,541	7	(12)
30-34 y	47	458	1,602,097	34	(15)	47	35	400,269	8	(11)
35-39 y	47	551	1,347,402	46	(20)	47	64	438,961	14	(15)
40-44 y	47	711	1,162,462	66	(27)	47	89	495,564	17	(17)
45-49 y	47	1,081	1,116,891	104	(36)	47	148	558,717	27	(20)
50-54 y	47	1,851	1,363,578	147	(34)	47	272	717,540	38	(20)
55-59 y	47	3,007	1,552,005	211	(51)	47	386	810,002	45	(22)
60-64 y	47	2,180	988,266	239	(68)	47	292	494,938	60	(39)
65-69 y	47	1,539	539,445	328	(109)	47	227	248,533	95	(64)
70-74 y	47	1,206	240,558	548	(196)	47	212	101,281	217	(158)
≥75 y	47	1,815	101,972	1,932	(581)	47	903	50,606	1,796	(907)
Workers not classifiable by occupation										
25-29 y	47	127	78,893	308	(362)	47	36	55,954	71	(173)
30-34 y	47	174	84,184	240	(249)	47	58	51,737	147	(299)
35-39 y	47	204	70,053	453	(506)	47	54	41,682	176	(450)
40-44 y	47	290	58,040	677	(617)	47	69	37,312	248	(351)
45-49 y	47	404	48,087	1,271	(1,396)	47	121	33,886	424	(623)
50-54 y	47	681	53,800	1,945	(1,723)	47	212	36,748	694	(569)
55-59 y	47	1,152	62,330	2,475	(2,020)	47	301	40,653	1,010	(1,485)
60-64 y	47	872	47,280	2,521	(2,115)	47	227	29,294	856	(1,016)
65-69 y	47	704	35,454	3,006	(3,570)	47	225	20,672	1,703	(1,801)
70-74 y	47	650	23,193	4,029	(3,436)	47	230	13,134	2,412	(2,645)
≥75 y	47	1,708	21,111	11,995	(10,293)	47	1,451	15,408	12,889	(9,838)
Non-employed [c]										
25-29 y	47	972	488,448	239	(89)	47	722	1,609,250	51	(21)
30-34 y	47	1,324	308,217	474	(157)	47	1,240	1,960,279	69	(21)
35-39 y	47	1,745	233,319	751	(184)	47	1,512	1,668,783	98	(33)
40-44 y	47	2,407	210,705	1,124	(278)	47	2,118	1,281,777	186	(60)
45-49 y	47	3,667	271,894	1,347	(286)	47	3,120	1,376,644	276	(89)
50-54 y	47	7,450	355,755	2,181	(383)	47	5,906	1,824,676	386	(103)
55-59 y	47	14,913	426,923	3,530	(455)	47	10,416	1,946,623	593	(93)
60-64 y	47	26,466	1,264,350	2,052	(281)	47	13,993	2,473,056	558	(78)
65-69 y	47	39,050	1,676,000	2,200	(287)	47	20,546	2,803,620	704	(90)
70-74 y	47	60,790	1,710,442	3,405	(333)	47	34,281	2,665,057	1,232	(128)
≥75 y	47	263,183	2,502,572	10,453	(620)	47	332,002	5,331,555	6,211	(355)

SD; standard deviation

[a] These cells are cross-classified by sex, age (5 year categories), and 11 occupations.

[b] Mortality rate was calculated on the basis of the means of the proportion of deaths for each cell type across all prefectures.

[c] Non-employed includes the unemployed as well as the non-labor force.
