# Supplementary material for: Geographic Inequalities in All-Cause Mortality in Japan: Compositional or Contextual?
Source: PLoS One. 2012 Jun 27;7(6):e39876. doi: 10.1371/journal.pone.0039876 (PMC3384616; doi:10.1371/journal.pone.0039876)
Supplement: Table S3 — Prefecture-level residuals for all-cause mortality by occupations among men, Japan, 2005. (PDF) [file pone.0039876.s007.pdf]

Table S3. Prefecture-level residuals for all-cause mortality by occupations among men, Japan, 2005

Rank [a]	Clerical, technical and managerial occupations		Sales and service occupations		Agriculture, forestry and fishery occupations		Production and transport occupations		Unclassifiable occupations		Non-employed [b]	
	No.	Prefecture	No.	Prefecture	No.	Prefecture	No.	Prefecture	No.	Prefecture	No.	Prefecture
1	27	Osaka	27	Osaka	15	Niigata	27	Osaka	43	Kumamoto	47	Okinawa
2	14	Kanagawa	11	Saitama	16	Toyama	14	Kanagawa	17	Ishikawa	32	Shimane
3	11	Saitama	14	Kanagawa	43	Kumamoto	34	Hiroshima	39	Kochi	33	Okayama
4	29	Nara	34	Hiroshima	18	Fukui	11	Saitama	30	Wakayama	44	Oita
5	34	Hiroshima	29	Nara	6	Yamagata	29	Nara	12	Chiba	29	Nara
6	13	Tokyo	13	Tokyo	17	Ishikawa	28	Hyogo	26	Kyoto	43	Kumamoto
7	12	Chiba	28	Hyogo	44	Oita	40	Fukuoka	24	Mie	25	Shiga
8	47	Okinawa	12	Chiba	29	Nara	44	Oita	8	Ibaraki	45	Miyazaki
9	28	Hyogo	40	Fukuoka	32	Shimane	43	Kumamoto	18	Fukui	34	Hiroshima
10	40	Fukuoka	44	Oita	7	Fukushima	13	Tokyo	2	Aomori	46	Kagoshima
11	33	Okayama	33	Okayama	46	Kagoshima	16	Toyama	37	Kagawa	20	Nagano
12	44	Oita	47	Okinawa	27	Osaka	32	Shimane	1	Hokkaido	38	Ehime
13	45	Miyazaki	4	Miyagi	45	Miyazaki	12	Chiba	14	Kanagawa	6	Yamagata
14	4	Miyagi	16	Toyama	25	Shiga	33	Okayama	22	Shizuoka	36	Tokushima
15	43	Kumamoto	43	Kumamoto	30	Wakayama	18	Fukui	45	Miyazaki	42	Nagasaki
16	38	Ehime	45	Miyazaki	20	Nagano	26	Kyoto	13	Tokyo	10	Gunma
17	41	Saga	17	Ishikawa	34	Hiroshima	25	Shiga	23	Aichi	41	Saga
18	17	Ishikawa	32	Shimane	22	Shizuoka	46	Kagoshima	34	Hiroshima	15	Niigata
19	32	Shimane	46	Kagoshima	19	Yamanashi	19	Yamanashi	20	Nagano	40	Fukuoka
20	25	Shiga	1	Hokkaido	23	Aichi	1	Hokkaido	33	Okayama	5	Akita
21	46	Kagoshima	18	Fukui	26	Kyoto	20	Nagano	40	Fukuoka	14	Kanagawa
22	16	Toyama	26	Kyoto	35	Yamaguchi	35	Yamaguchi	25	Shiga	39	Kochi
23	1	Hokkaido	19	Yamanashi	37	Kagawa	17	Ishikawa	7	Fukushima	24	Mie
24	15	Niigata	25	Shiga	14	Kanagawa	47	Okinawa	38	Ehime	18	Fukui
25	31	Tottori	35	Yamaguchi	40	Fukuoka	45	Miyazaki	11	Saitama	28	Hyogo
26	18	Fukui	15	Niigata	47	Okinawa	4	Miyagi	5	Akita	4	Miyagi
27	26	Kyoto	38	Ehime	42	Nagasaki	22	Shizuoka	28	Hyogo	16	Toyama
28	19	Yamanashi	31	Tottori	41	Saga	15	Niigata	4	Miyagi	22	Shizuoka
29	9	Tochigi	37	Kagawa	1	Hokkaido	37	Kagawa	9	Tochigi	21	Gifu
30	10	Gunma	20	Nagano	33	Okayama	21	Gifu	15	Niigata	1	Hokkaido
31	35	Yamaguchi	22	Shizuoka	38	Ehime	38	Ehime	36	Tokushima	19	Yamanashi
32	37	Kagawa	41	Saga	31	Tottori	9	Tochigi	44	Oita	31	Tottori
33	6	Yamagata	9	Tochigi	39	Kochi	24	Mie	31	Tottori	35	Yamaguchi
34	20	Nagano	6	Yamagata	4	Miyagi	6	Yamagata	47	Okinawa	9	Tochigi
35	7	Fukushima	21	Gifu	13	Tokyo	42	Nagasaki	29	Nara	11	Saitama
36	22	Shizuoka	7	Fukushima	12	Chiba	10	Gunma	35	Yamaguchi	37	Kagawa
37	36	Tokushima	10	Gunma	28	Hyogo	31	Tottori	27	Osaka	17	Ishikawa
38	21	Gifu	23	Aichi	21	Gifu	23	Aichi	42	Nagasaki	8	Ibaraki
39	8	Ibaraki	42	Nagasaki	10	Gunma	7	Fukushima	16	Toyama	27	Osaka
40	23	Aichi	8	Ibaraki	24	Mie	41	Saga	19	Yamanashi	30	Wakayama
41	42	Nagasaki	24	Mie	9	Tochigi	39	Kochi	6	Yamagata	26	Kyoto
42	3	Iwate	36	Tokushima	11	Saitama	8	Ibaraki	3	Iwate	3	Iwate
43	39	Kochi	3	Iwate	8	Ibaraki	36	Tokushima	10	Gunma	23	Aichi
44	24	Mie	39	Kochi	5	Akita	30	Wakayama	21	Gifu	7	Fukushima
45	30	Wakayama	2	Aomori	2	Aomori	5	Akita	32	Shimane	12	Chiba
46	2	Aomori	30	Wakayama	36	Tokushima	3	Iwate	46	Kagoshima	13	Tokyo
47	5	Akita	5	Akita	3	Iwate	2	Aomori	41	Saga	2	Aomori

Prefectures with significantly low and high odds for all-cause mortality are shown in blue and red, respectively.

[a] Prefectures with a lower estimate of odds for all-cause mortality are ranked higher. The reference is the grand mean of all prefectures.

[b] Non-employed includes the unemployed as well as the non-labor force.
