# Supplementary material for: Geographic Inequalities in All-Cause Mortality in Japan: Compositional or Contextual?
Source: PLoS One. 2012 Jun 27;7(6):e39876. doi: 10.1371/journal.pone.0039876 (PMC3384616; doi:10.1371/journal.pone.0039876)
Supplement: Table S4 — Prefecture-level residuals for all-cause mortality by occupations among women, Japan, 2005. (PDF) [file pone.0039876.s008.pdf]

Table S4. Prefecture-level residuals for all-cause mortality by occupations among women, Japan, 2005

Rank [a]	Clerical, technical and managerial occupations		Sales and service occupations		Agriculture, forestry and fishery occupations		Production and transport occupations		Unclassifiable occupations		Non-employed [b]	
	No.	Prefecture	No.	Prefecture	No.	Prefecture	No.	Prefecture	No.	Prefecture	No.	Prefecture
1	29	Nara	29	Nara	15	Niigata	29	Nara	2	Aomori	32	Shimane
2	27	Osaka	27	Osaka	32	Shimane	18	Fukui	12	Chiba	47	Okinawa
3	41	Saga	34	Hiroshima	6	Yamagata	31	Tottori	30	Wakayama	33	Okayama
4	31	Tottori	31	Tottori	41	Saga	27	Osaka	20	Nagano	43	Kumamoto
5	34	Hiroshima	18	Fukui	7	Fukushima	17	Ishikawa	5	Akita	34	Hiroshima
6	47	Okinawa	44	Oita	19	Yamanashi	15	Niigata	17	Ishikawa	29	Nara
7	6	Yamagata	47	Okinawa	16	Toyama	44	Oita	26	Kyoto	44	Oita
8	4	Miyagi	41	Saga	22	Shizuoka	34	Hiroshima	15	Niigata	46	Kagoshima
9	44	Oita	28	Hyogo	20	Nagano	6	Yamagata	39	Kochi	14	Kanagawa
10	19	Yamanashi	6	Yamagata	29	Nara	26	Kyoto	18	Fukui	15	Niigata
11	33	Okayama	19	Yamanashi	35	Yamaguchi	19	Yamanashi	22	Shizuoka	45	Miyazaki
12	18	Fukui	4	Miyagi	25	Shiga	16	Toyama	8	Ibaraki	31	Tottori
13	11	Saitama	32	Shimane	21	Gifu	28	Hyogo	1	Hokkaido	40	Fukuoka
14	32	Shimane	17	Ishikawa	31	Tottori	20	Nagano	23	Aichi	42	Nagasaki
15	28	Hyogo	33	Okayama	17	Ishikawa	25	Shiga	9	Tochigi	6	Yamagata
16	40	Fukuoka	15	Niigata	33	Okayama	12	Chiba	24	Mie	41	Saga
17	17	Ishikawa	40	Fukuoka	28	Hyogo	47	Okinawa	38	Ehime	25	Shiga
18	35	Yamaguchi	35	Yamaguchi	18	Fukui	14	Kanagawa	37	Kagawa	20	Nagano
19	15	Niigata	11	Saitama	34	Hiroshima	32	Shimane	13	Tokyo	1	Hokkaido
20	21	Gifu	16	Toyama	30	Wakayama	33	Okayama	25	Shiga	18	Fukui
21	16	Toyama	14	Kanagawa	26	Kyoto	41	Saga	40	Fukuoka	11	Saitama
22	46	Kagoshima	25	Shiga	43	Kumamoto	37	Kagawa	14	Kanagawa	27	Osaka
23	43	Kumamoto	21	Gifu	37	Kagawa	4	Miyagi	7	Fukushima	28	Hyogo
24	14	Kanagawa	46	Kagoshima	5	Akita	24	Mie	34	Hiroshima	39	Kochi
25	45	Miyazaki	12	Chiba	3	Iwate	46	Kagoshima	44	Oita	19	Yamanashi
26	10	Gunma	26	Kyoto	9	Tochigi	35	Yamaguchi	11	Saitama	4	Miyagi
27	13	Tokyo	20	Nagano	42	Nagasaki	2	Aomori	28	Hyogo	16	Toyama
28	9	Tochigi	37	Kagawa	11	Saitama	1	Hokkaido	43	Kumamoto	5	Akita
29	25	Shiga	45	Miyazaki	23	Aichi	21	Gifu	31	Tottori	10	Gunma
30	26	Kyoto	9	Tochigi	14	Kanagawa	40	Fukuoka	4	Miyagi	22	Shizuoka
31	37	Kagawa	10	Gunma	38	Ehime	9	Tochigi	33	Okayama	17	Ishikawa
32	30	Wakayama	30	Wakayama	39	Kochi	30	Wakayama	35	Yamaguchi	38	Ehime
33	12	Chiba	24	Mie	10	Gunma	11	Saitama	45	Miyazaki	35	Yamaguchi
34	20	Nagano	1	Hokkaido	4	Miyagi	22	Shizuoka	6	Yamagata	13	Tokyo
35	22	Shizuoka	43	Kumamoto	44	Oita	7	Fukushima	16	Toyama	7	Fukushima
36	38	Ehime	7	Fukushima	45	Miyazaki	5	Akita	29	Nara	37	Kagawa
37	42	Nagasaki	13	Tokyo	24	Mie	45	Miyazaki	3	Iwate	36	Tokushima
38	24	Mie	22	Shizuoka	27	Osaka	43	Kumamoto	32	Shimane	24	Mie
39	7	Fukushima	38	Ehime	46	Kagoshima	39	Kochi	19	Yamanashi	26	Kyoto
40	3	Iwate	2	Aomori	8	Ibaraki	38	Ehime	42	Nagasaki	21	Gifu
41	5	Akita	5	Akita	12	Chiba	3	Iwate	47	Okinawa	12	Chiba
42	1	Hokkaido	3	Iwate	40	Fukuoka	23	Aichi	27	Osaka	3	Iwate
43	8	Ibaraki	42	Nagasaki	36	Tokushima	13	Tokyo	46	Kagoshima	8	Ibaraki
44	2	Aomori	39	Kochi	2	Aomori	10	Gunma	36	Tokushima	9	Tochigi
45	36	Tokushima	8	Ibaraki	1	Hokkaido	8	Ibaraki	10	Gunma	30	Wakayama
46	39	Kochi	36	Tokushima	47	Okinawa	42	Nagasaki	21	Gifu	2	Aomori
47	23	Aichi	23	Aichi	13	Tokyo	36	Tokushima	41	Saga	23	Aichi

Prefectures with significantly low and high odds for all-cause mortality are shown in blue and red, respectively.

[a] Prefectures with a lower estimate of odds for all-cause mortality are ranked higher. The reference is the grand mean of all prefectures.

[b] Non-employed includes the unemployed as well as the non-labor force.
