# Supplementary material for: Geographic Inequalities in All-Cause Mortality in Japan: Compositional or Contextual?
Source: PLoS One. 2012 Jun 27;7(6):e39876. doi: 10.1371/journal.pone.0039876 (PMC3384616; doi:10.1371/journal.pone.0039876)
Supplement: Table S5 — Variance and covariance matrices of prefecture-level variances of each occupation group, Japan, 2005. (PDF) [file pone.0039876.s009.pdf]

Table S5. Variance and covariance matrices of prefecture-level variances of each occupation group, Japan, 2005 [a]

	Men						Women					
	Clerical, technical and managerial occupations	Sales and service occupations	Agriculture, forestry and fishery occupations	Production and transport occupations	Unclassifiable occupations	Non-employed [b]	Clerical, technical and managerial occupations	Sales and service occupations	Agriculture, forestry and fishery occupations	Production and transport occupations	Unclassifiable occupations	Non-employed [b]
Clerical, technical and managerial occupations	0.038						0.016					
	(0.009)						(0.004)					
	1.000						*1.000*					
Sales and service occupations	0.039	0.044					0.018	0.027				
	(0.009)	(0.010)					(0.005)	(0.007)				
	0.944	*1.000*					*0.856*	*1.000*				
Agriculture, forestry and fishery occupations	0.006	0.008	0.023				0.006	0.011	0.055			
	(0.005)	(0.005)	(0.006)				(0.006)	(0.007)	(0.014)			
	0.201	*0.259*	*1.000*				*0.217*	*0.273*	*1.000*			
Production and transport occupations	0.028	0.033	0.010	0.031			0.016	0.030	0.019	0.055		
	(0.007)	(0.008)	(0.005)	(0.007)			(0.006)	(0.009)	(0.011)	(0.016)		
	0.807	*0.911*	*0.371*	*1.000*			*0.538*	*0.777*	*0.356*	*1.000*		
Unclassifiable occupations	0.004	0.003	0.006	0.008	0.550		-0.034	-0.029	-0.021	0.017	0.515	
	(0.023)	(0.024)	(0.018)	(0.021)	(0.125)		(0.017)	(0.021)	(0.029)	(0.031)	(0.120)	
	0.025	*0.016*	*0.064*	*0.064*	*1.000*		*-0.379*	*-0.248*	*-0.123*	*0.102*	*1.000*	
Non-employed [b]	0.004	0.002	0.0030	0.003	-0.013	0.005	0.004	0.005	0.003	0.005	-0.015	0.004
	(0.002)	(0.002)	(0.002)	(0.002)	(0.009)	(0.001)	0.002	(0.002)	(0.002)	(0.003)	(0.008)	(0.001)
	0.250	*0.153*	*0.233*	*0.233*	*-0.245*	*1.000*	*0.540*	*0.502*	*0.196*	*0.321*	*-0.320*	*1.000*

[a] The number in parentheses is a standard error of the corresponding variances and covariances. The italicized numbers are correlation coefficients.

[b] Non-employed includes the unemployed as well as the non-labor force.
