# Supplementary material for: Geographic Inequalities in All-Cause Mortality in Japan: Compositional or Contextual?
Source: PLoS One. 2012 Jun 27;7(6):e39876. doi: 10.1371/journal.pone.0039876 (PMC3384616; doi:10.1371/journal.pone.0039876)
Supplement: Table S6 — Predicted number of all-cause mortality (per 100,000) by each occupation group, Japan, 2005. (PDF) [file pone.0039876.s010.pdf]

Table S6. Predicted number of all-cause mortality (per 100,000) by each occupation group, Japan, 2005 [a]

	Men		Women	
Occupation	Estimate	95% CI	Estimate	95% CI
Clerical, technical and managerial occupations	612	583, 643	155	148, 162
Sales and service occupations	594	564, 627	161	153, 170
Agriculture, forestry and fishery occupations	781	745, 820	164	153, 178
Production and transport occupations	289	275, 302	80	74, 87
Unclassifiable occupations	2,790	2,258, 3,463	1,044	858, 1,271
Non-employed [b]	2,264	2,210, 2,322	478	467, 490

CI; credible interval

[a] We calculated these estimates by using mean predicted probabilities for all-cause mortality among those aged 55 to 59.

[b] Non-employed includes the unemployed as well as the non-labor force.
